# Monolayer-Graphene-Based Tunable Absorber in the Near-Infrared

**DOI:** 10.3390/mi12111320

**Published:** 2021-10-28

**Authors:** Shuhua Cao, Qi Wang, Xufeng Gao, Shijie Zhang, Ruijin Hong, Dawei Zhang

**Affiliations:** Engineering Research Center of Optical Instrument and System, Ministry of Education and Shanghai Key Laboratory of Modern Optical System, School of Optical-Electrical and Computer Engineering, University of Shanghai for Science and Technology, 516 Jungong Rd, Shanghai 200093, China; 181390037@st.usst.edu.cn (S.C.); 192380285@st.usst.edu.cn (X.G.); 202310334@st.usst.edu.cn (S.Z.); rjhong@usst.edu.cn (R.H.); dwzhang@usst.edu.cn (D.Z.)

**Keywords:** graphene, absorber, Fano resonance, Fermi energy, sensor

## Abstract

In this paper, a tunable absorber composed of asymmetric grating based on a graphene-dielectric-metal structure is proposed. The absorption of the absorber can be modified from 99.99% to 61.73% in the near-infrared by varying the Fermi energy of graphene, and the absorption wavelength can be tuned by varying the grating period. Furthermore, the influence of other geometrical parameters, the incident angle, and polarization are analyzed in detail by a finite-difference time-domain simulation. The graphene absorbers proposed in this paper have potential applications in the fields of stealth, sense, and photoelectric conversion. When the absorber that we propose is used as a gas sensor, the sensitivity of 200 nm/RIU with FOM can reach up to 159 RIU^−1^.

## 1. Introduction

Graphene can absorb light over a broad spectrum spanning from the ultraviolet to the terahertz spectral regime due to its gapless nature and its ability to modulate the absorption by controlling the inter-band and intra-band transitions [1]. Therefore, graphene is an ideal material for optoelectronic devices such as photo-detectors [2], filters [3], switches [4], sensors [5,6], and modulators [7]. However, the absorption of monolayer graphene is only 2.3%, which is far from meeting the requirements of optoelectronic devices. Recently, numerous near-infrared absorbers using graphene and silicon gratings have been proposed. Akhavan et al. designed a graphene absorber, the efficient absorption of light by a graphene sheet was realized by guided mode resonance [8]. Zheng et al. designed an absorber with a high absorption efficiency at an incident angle of 0 to 5 degrees by using Fabry-Perot cavity resonance [9]. Hu et al. proposed a multilayer subwavelength grating structure to adjust the absorption efficiency by varying the incident angle [10]. Hence, the focus of research is to improve the absorption of graphene devices via various resonance effects, such as guided-mode resonance [1], surface plasmon resonance [11], Fabry-Perot resonance [12], Fano resonance [13], among others.

Fano resonance generates a large electromagnetic field in and around its structure, exhibiting a sharp asymmetric peak [14], which occurs when a narrow dark mode weakly couples to a wide bright mode [15,16]. In recent years, Fano resonance has been realized for different types of micro-nano structures ranging from visible light to far-infrared, and many efforts have been devoted to its tunability and high efficiency.

In this paper, the proposed absorber is composed of asymmetric periodic grating. causing the asymmetry absorption spectrum. Demonstrated by the finite-difference time-domain (FDTD) simulations, it shows that the absorption can be tuned by varying the Fermi energy of graphene in the structure. In addition, the influences of period, groove depth, incident angle, and polarization on absorption are also studied. The period is the main factor affecting the resonance wavelength and the absorber is proven to tolerate a wide range of incident angles from −15° to +15°. The proposed tunable absorber has broad application prospects in detectors, invisibility cloaking, sensors, filters, and energy harvesting. Therefore, we finally studied the proposed absorber as a gas sensor; and the sensitivity and FOM of the sensor can reach up to 200 nm/RIU and 159 RIU^−1^, respectively.

## 2. Structure and Simulation

**Figure 1:**
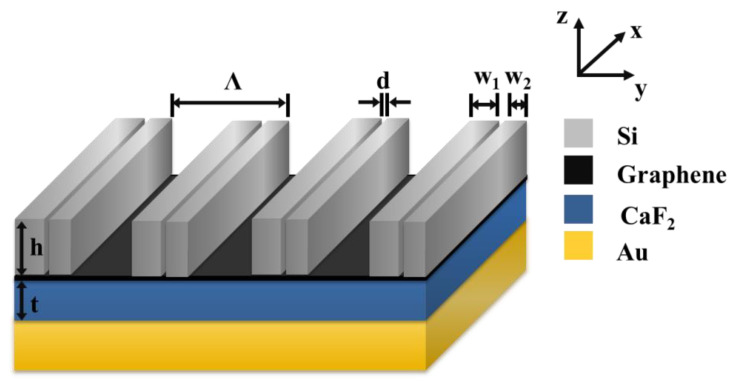
Schematic diagram of the proposed absorber.

Figure 1 presents the schematics of the proposed structure consisting of monolayer graphene sandwiched between silicon (Si) grating and calcium fluoride (CaF_2_) film, where h and t indicate groove depth of the Si grating and the CaF_2_ film layer, w_1_ and w_2_ are the widths of the two grating ridges in one grating period, d is the distance between the two grating ridges, and Λ is the grating period. Here, Si and CaF_2_ are assumed to be lossless and dispersion-free, with the refractive indexes are of 3.48 and 1.43, respectively. The gold (Au) film at the bottom of the structure needs to be thick enough to avoid the transmission of the incident light. The Au film used in the simulation is 300 nm thick, and its refractive index is from Palik’s handbook [17]. The background refractive index of the structure is assumed to be 1.00.

The graphene is modeled as a thin dielectric layer with a permittivity, and the permittivity of graphene is calculated by conductivity. Conductivity is calculated as a sum of the intra-band $\sigma_{\mathrm{intra}}$ and inter-band $\sigma_{\mathrm{inter}}$ conductivity:

(1) $$\sigma_{\mathrm{intra}}=\frac{ie^{2}k_{B}T}{\pi \hbar^{2}(\omega +i/\tau )}\left(\frac{E_{f}}{k_{B}T}+2\ln\left(e^{-\frac{E_{f}}{k_{B}T}}+1\right)\right)$$

(2) $$\sigma_{\mathrm{inter}}=\frac{ie^{2}}{4\pi \hbar }\ln\left(\frac{2E_{f}-(\omega +i/\tau )\hbar }{2E_{f}+(\omega +i/\tau )\hbar }\right)$$

(3) $$\sigma =\sigma_{\mathrm{intra}}+\sigma_{\mathrm{inter}}$$

where e is the electron charge, $k_{B}$ is the Boltzmann constant, T is the temperature, ℏ is the reduced Planck constant, ω is the angular frequency, $E_{f}$ is the Fermi energy, and τ is the carrier scattering time [18]. In our simulation, the initial Fermi energy of graphene is assumed to be $E_{f}=0.55\;\mathrm{eV}$, and the carrier scattering time is chosen as τ=0.5 ps.

The anisotropic relative permittivity $\varepsilon_{g\mathrm{raphene}}$ of graphene is calculated by the following formula:

(4) $$\varepsilon_{g\mathrm{raphene}}=\left(\begin{array}{ccc} 1+i\sigma /\left(\omega \varepsilon_{0}t_{g}\right) & 0 & 0\\ 0 & 1+i\sigma /\left(\omega \varepsilon_{0}t_{g}\right) & 0\\ 0 & 0 & 1 \end{array}\right)$$

where $\varepsilon_{0}$ is the permittivity in vacuum, and $t_{g}$ is the thickness of graphene, which is assumed to 0.34 nm in the calculation.

**Figure 2:**
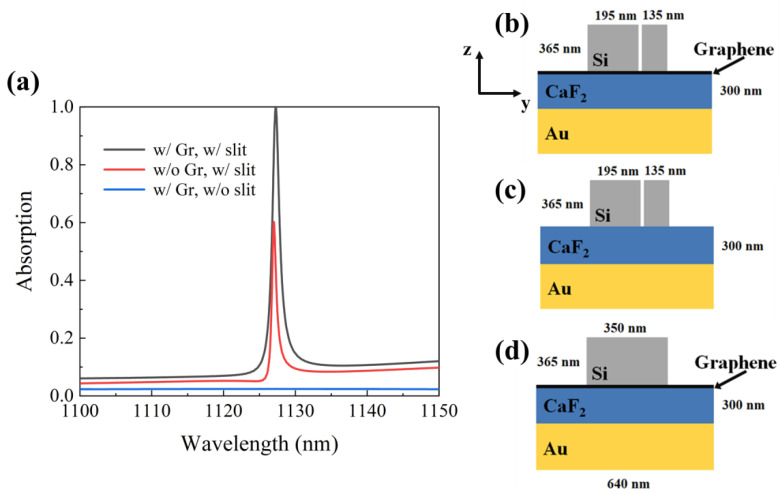
(**a**) Absorption spectrum at normal incidence for TE polarization at three different configurations. (**b**) The structure includes monolayer graphene and slit, and the corresponding absorption curve is black. (**c**) The structure does not include monolayer graphene but includes a slit, and the corresponding absorption curve is red. (**d**) The structure includes monolayer graphene but does not include a slit, and the corresponding absorption curve is blue.

The optimum structural parameters of the grating were chosen as follows: Λ = 640 nm, h = 365 nm, t = 300 nm, w_1_ = 195 nm, w_2_ = 135 nm, d = 20 nm. First of all, we discuss the influence of the existence of slit and monolayer graphene on the absorption spectrum. Under the incidence of TE polarized light, as shown in Figure 2a, when the structure contaions both graphene and slit, its absorption is as high as 99.99% at 1227.27 nm, and the schematic diagram of the structure is shown in Figure 2b. For the structure without graphene, its absorption is shown as the red curve in Figure 2a, and the absorption is only 60.23%, which schematic diagram of the structure is shown in Figure 2c. Even without graphene, the structure can still absorb part of the incident light. However, structures with and without slits differ greatly in the absorption. If there are no slits in the structure, as shown in Figure 2d, there will be no resonance peak in Figure 2a. This is because the presence of the slit makes the structure asymmetric, resulting in Fano resonance.

**Figure 3:**
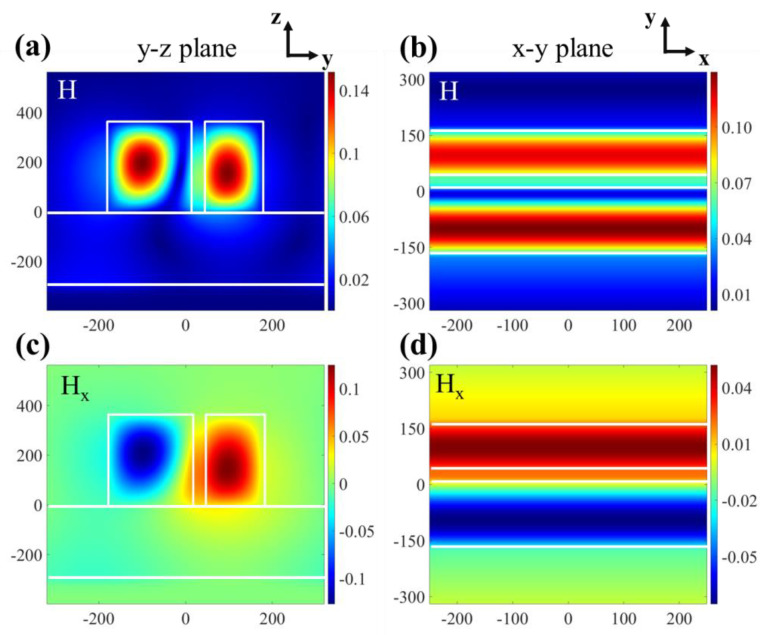
The magnetic field distribution at the peak of the Fano resonance spectral curve: (**a**) at y-z plane, (**b**) at x-y plane. The magnetic field in x-direction normalized to the field amplitude of the incident light: (**c**) at y-z plane, (**d**) at x-y plane.

The local electromagnetic field is the key physical process to generate Fano resonance, which can be achieved by the interaction of the excited modes. Si with a high refractive index is used as the ridge of our proposed grating. Therefore, when there are no slits in the structure, the optical properties of the grating are similar to those of the planar waveguide. At this time, the structure is symmetrical, so these modes are not coupled to the radiation modes. As shown in Figure 3a–d, when there are slits in the grating, the grating ridges are divided into two grating ridges of different widths. Then, the symmetry of the structure is destroyed, and the two grating ridges with different widths excite the reverse current distribution, thus forming magnetic dipoles perpendicular to the surface, generating a narrow-band sub-radiation mode, which is coupled with the radiation mode to form a Fano resonance.

**Figure 4:**
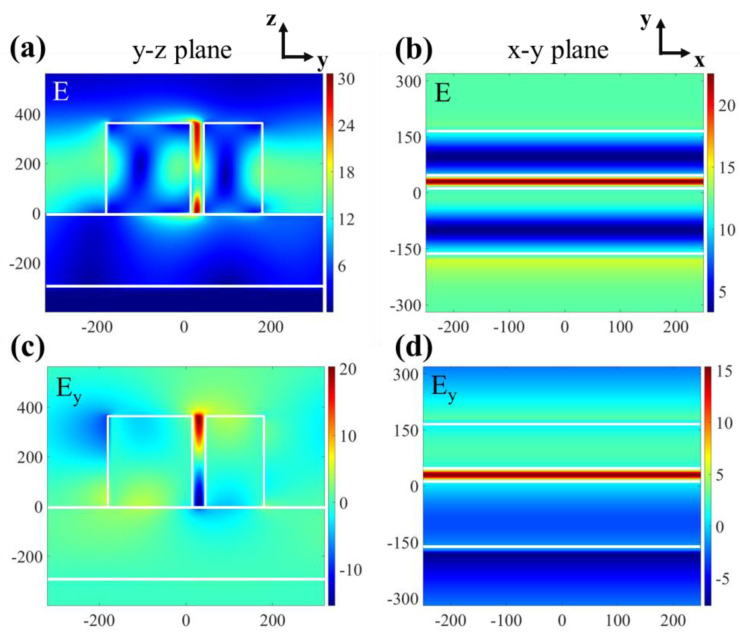
The electric field distribution at the peak of the Fano resonance spectral curve, (**a**) at y-z plane, (**b**) at x-y plane. The electric field in y-direction normalized to the field amplitude of the incident light, (**c**) at y-z plane, (**d**) at x-y plane.

The electric field distribution at the resonance wavelength is also calculated and reported in Figure 4a–d. Clearly, a large electric field enhancement appears within the slit due to the Fano resonance. The maximum enhancement of the field amplitude is about 30 (see Figure 4a). When different materials surround or are embedded in this structure, the combination of a sharp absorption response and enlarged fields is ideal for achieving stronger absorption sensitivity. Therefore, the perfect absorber we proposed can be operated as a refractive index sensor.

**Figure 5:**
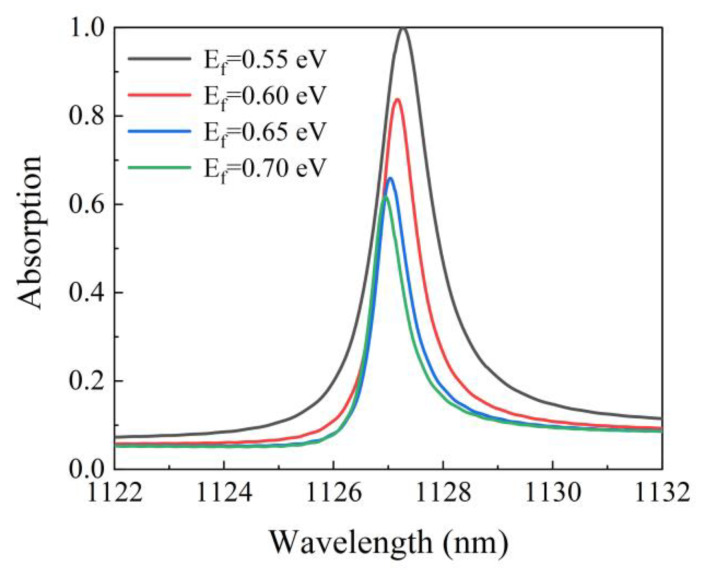
The changes in the absorption spectrum of the structure with the increase in Fermi energy, and the other parameters are kept constant.

Figure 5 clearly illustrates the relationship between the Fermi energy of graphene and absorption. It is seen that when the Fermi energy increases from 0.55 eV to 0.70 eV, the resonance wavelength shifts from 1127.27 nm to 1126.95 nm, and the absorption decreases from 99.99% to 61.73%. The reason for this blue shift is that an increase in the Fermi energy of the monolayer graphene requires a high energy to induce the resonance between the generated electron and the incident electromagnetic wave. This higher energy requirement results in a decrease in the effective resonance wavelength. Meanwhile, with the increase in Fermi energy, the imaginary part of the permittivity decreases monotonically, which leads to the decrease in absorption. The Fermi energy of graphene increases as the gate voltage increases. Therefore, the tuning of absorption can be conveniently achieved by controlling the gate voltage.

**Figure 6:**
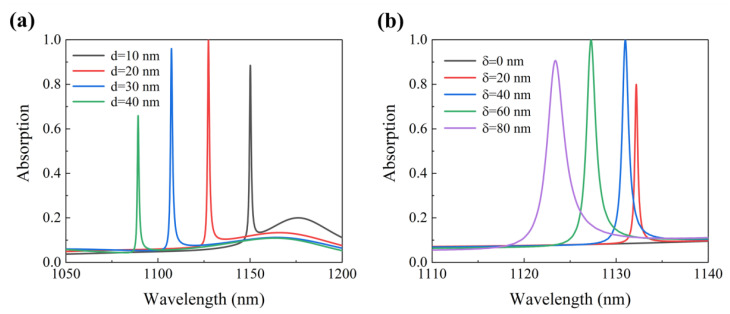
(**a**) Absorption spectrum for different slit widths. (**b**) Absorption spectrum for different asymmetry parameters.

Figure 6a shows the effects of the slit width on the absorption of the structure. The resonance wavelength shows a blueshift with a slit width that increased from 10 nm to 40 nm, whereas the absorption first increased and then decreased. Moreover, when the width of the slit is 20 nm, the maximum absorption of the structure is 99.99%. It can be seen from the figure that the optimal distance d between the two grating ridges is 20 nm. When d is longer than the optimal distance, the interaction between the two grating ridges decreases, resulting in the a decrease in absorption.

The asymmetry parameter δ is defined as the difference in the grating ridges widths $\delta =w_{1}-w_{2}$. As shown in Figure 6b, When δ=0 nm ($w_{1}=w_{2}=165\;\mathrm{nm}$), there is no absorption in the spectrum. This is because there is only one slit in a period of the structure, the width of the two grating ridges is the same, and the structure is still symmetrical, so the Fano resonance is not excited. When the δ increases from 40 nm ($w_{1}=185\;\mathrm{nm},\;w_{2}=145\;\mathrm{nm}$) to 60 nm ($w_{1}=195\;\mathrm{nm},\;w_{2}=135\;\mathrm{nm}$), the resonance wavelength shifts from 1131.01 nm to 1127.27 nm where the absorption increases from 99.86% to 99.99%. Therefore, we choose a width difference of 60 nm between the two grating ridges.

**Figure 7:**
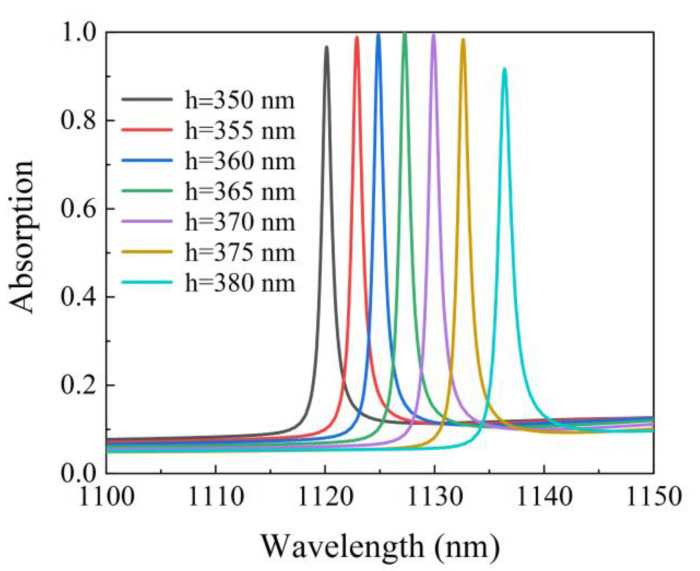
Absorption spectrum for different groove depths of Si grating.

Figure 7 illustrates the relationship between absorption spectrum versus the wavelength with different groove depths of the Si grating. When the groove depths of Si grating h are changed from 350 nm to 380 nm, the resonance wavelength of the proposed structure shows a redshift. This is because the equivalent optical thickness of the structure increases with the increase in groove depth of the Si grating. In addition, altering the groove depth of the Si grating has a negligible effect on the FWHM of the absorption spectrum. Therefore, the resonance wavelength can be tuned in the near-infrared by adjusting a suitable groove depth. Due to the high absorption capacity of the proposed structure, a large error tolerance can be maintained for the fabrication process imperfections of the groove depth of the Si grating.

**Figure 8:**
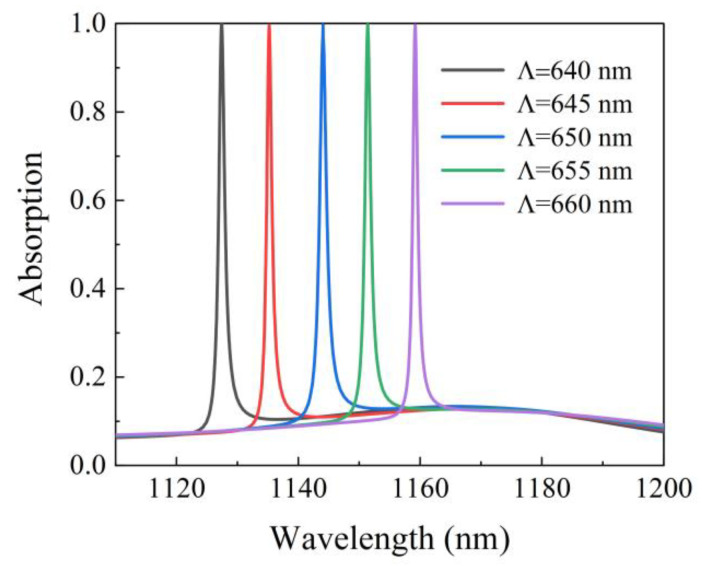
Changes in absorption while varying the period of the absorber structure.

As is shown in Figure 8, with the increase in the period Λ from 640 nm to 660 nm, the redshift will occur for the resonance wavelength, which results from the increase in the effective refractive index of the grating as the period rises. In addition, when the resonance wavelength is redshifted, the absorption is maintained at more than 99%. It is very significant that the enhanced absorption performance can be maintained in a wide wavelength range. Therefore, the absorption wavelength of the absorber can be linearly tuned by changing the grating period.

**Figure 9:**
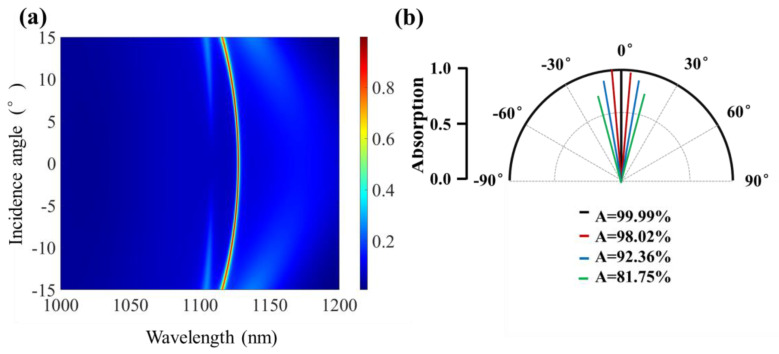
(**a**) Absorption spectrum of the graphene absorber versus the angle of incidence and wavelength. (**b**) Absorption angular pattern at seven different incident angles θ=0°,±5°,±10°,±15°.

The above discussion is based on normal incident light, but in the application of photonic devices, the proposed absorber should work in a wide range of light incident angles to ensure a high optical absorption efficiency. To study the angular sensitivity of the absorber, the absorption as a function of angle of incidence and wavelength is shown in Figure 9a. It can be found that the resonance wavelength blueshifts with varing the incident angle and the absorption is also changed with the variation of the incident angle. A polar plot of the absorption at the incident angles of θ=0°, θ=±5°, θ=±10°, θ=±15° is shown in Figure 9b. It can be seen that their corresponding peak absorptions are 99.99%, 98.02%, 92.36%, 81.75%, respectively. It is clear that the absorption peak slightly decreases with the increase in the angle of incidence. However, there is a maximum absorption greater than 80% when the incident angle increases to ±15°. Obviously, the proposed absorber can tolerate a wide range of incident angles.

.

**Figure 10:**
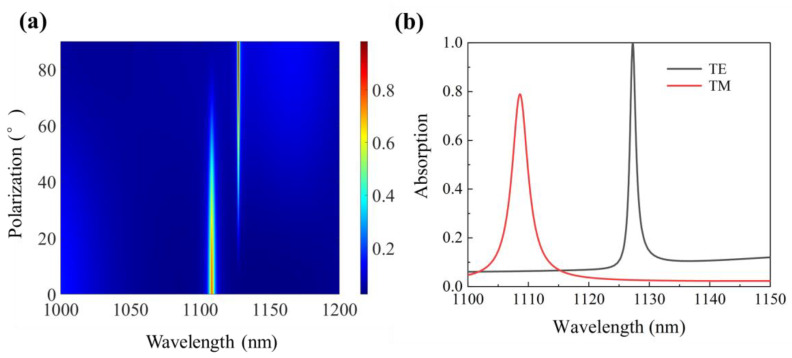
(**a**) Polarization dependent light absorption spectrum of the absorber. (**b**) Absorption of the proposed absorber under the illumination of TE and TM polarized normal incident light.

Because of the nature of the enhancement mechanism, the absorber is also sensitive to the polarization of the incident light. The polarization-dependent light absorption spectrum of the structure is shown in Figure 10a, and the absorption peak value at 1127.27 nm is weakened by the polarization of the polarization from TE to TM. The intensity of the absorption peak is different under the illumination of TE and TM polarized normal incident light. As shown in Figure 10b, with the polarization changes from TE to TM, the resonance wavelength shifts from 1127.27 nm to 1108.59 nm where the absorption decreases from 99.99% to 78.88%. It can be observed that the polarization of the incident light leads to a decrease in the absorption of the TM, compared to the TE polarization. This effect can be attributed to the propagation of the electric field for each polarization, i.e., the TE polarization electric field induces a higher charge displacement in the graphene sheets, due to its parallel orientation with respect to the surface, as compared to TM polarization, where a part of the electric field can propagate loosely (perpendicular part of the electric field) and the rest is absorbed (parallel part of the electric field) [19].

**Figure 11:**
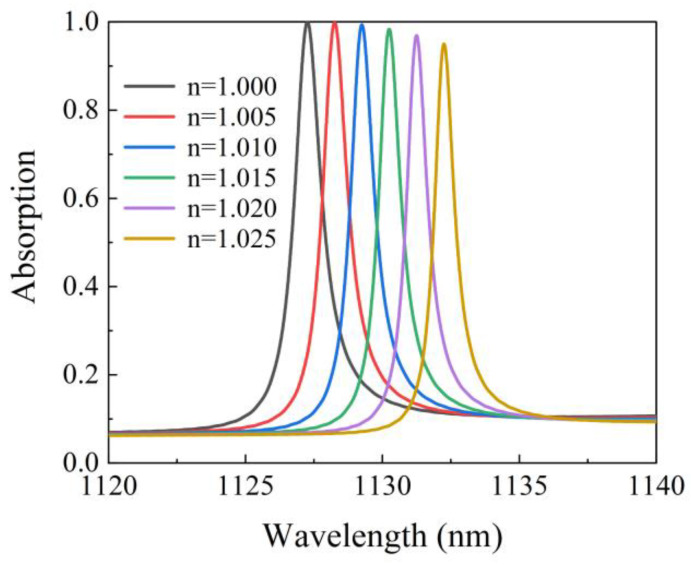
Absorption spectrum as a function of the refractive index of the background.

When our proposed absorber is used as a sensor, the grating surface is covered with gases with a refractive index range from 1.000 to 1.025. The positions of the resonance wavelength are plotted together as a function of the refractive index of the environment medium as shown in Figure 11, exhibiting a linear change in the wavelength shift. Sensitivity is an important indicator to evaluate the sensor quality, which is defined as the change in the resonance wavelength per refractive index unit [20]. By linearly fitting the data in Figure 11, we can obtain the sensitivity S = 200 nm/RIU. The figure of merit (FOM = S/FWHM) also reflects the sensing performance directly, which strongly depends on the resonant bandwidth. For the ultra-narrow band (FWHM = 1.26 nm), the FOM is 159 RIU^−1^. Based on the above results, the potential applications of the absorbers as sensors can provide useful insights in future applications.

Table 1 presents a comparison of the sensitivity and FOM of our work and the previously published refractive index sensors. We can see that our structure improves both sensitivity and FOM. Thus, these absorbers are ideal for applications in sensing.

## 3. Conclusions

In this paper, we proposed a type of absorber composed of asymmetric periodic gratings. A large field enhancement and optical localization can be realized in the slit between the two grating ridges with different widths. Fano resonance is generated by the asymmetry of structure. By optimizing the structure, we can obtain a perfect absorber with a narrow bandwidth, which has an absorption reaching up to 99.99% at 1127.27 nm when the Fermi energy of graphene is 0.55 eV. Moreover, the simulation results demonstrated that the absorption resonance peak and operating wavelength can be tuned by varying the Fermi energy of graphene and the geometrical parameters, as well as the incident angle and the polarization of light. This absorber can be applied in the design of optoelectronic devices, filters, and sensors. In the extended design of the proposed absorber, the gas sensor obtained a sensitivity as high as S = 200 nm/RIU with FOM = 159 RIU^−1^.

## Figures and Tables

**Table 1:** Comparison on sensitivity and FOM between this work and previous works.

Reference	Structure	S (nm/RIU)	FOM (RIU^−1^)
Yan et al. [21]	Al-Al_2_O_3_-graphene-Al_2_O_3_ grating	150	50
Varshney et al. [22]	Graphene metamaterial	-	53.09
Imas et al. [23]	D-shaped fiber	40	114
Lu et al. [24]	Glass-Au-SiO_2_-Au grating	190	25
Zhang et al. [25]	Dielectric ring metamaterial	104	21.8
Qian et al. [26]	SiO_2_-Ta_2_O_5_ grating	125.89	8.99
This work	Asymmetric grating	200	159
